# Rights, not rescue: trafficking (in)securities at the sport mega-event

**DOI:** 10.3389/fspor.2023.1207595

**Published:** 2023-09-11

**Authors:** Benton J. Oliver, Amanda De Lisio

**Affiliations:** School of Kinesiology and Health Science, York University, Toronto, ON, Canada

**Keywords:** anti-trafficking, coloniality, policing, sex work, FIFA world cup, sport mega-event

## Abstract

We examine the impact of fantasies used in the redevelopment of sport mega-event cities on host communities; particularly as related to the male-dominated FIFA World Cup and forced prostitution. We start with a discussion of event fantasies, particularly those that circulate in relation to humanitarian aid and the alleged involvement of women and children in forced labour and sexual exploitation. We trace these fantasies across several FIFA host cities since the 2006 FIFA World Cup, hosted in Germany, to leverage continual and perpetuate attention (and profit) through the non-profit industrial complex. These fantasies have facilitated and coordinated collaborative consensus amongst state authorities and allies to act in a meaningful manner even as the evidence of forced prostitution is still scant—while the realities of people that continue to be subjected to violent and exploitative labour in the construction of stadia, athlete recruitment, or equipment and apparel industries are seldom addressed. We do this to question the lived impact of policies and personalities of rescue on people engaged, consensually, in erotic labour within host cities, that are often made target of rescue intervention. The figure of the proverbial sex slave, as a highly racialized and hypersexualized trope, is mobilized through the sport mega-event to further police the bodies of all women in labour and migration. We end with a cautious message to future host cities, particularly cities implicated in the 2026 FIFA World Cup within Mexico, Canada, and the United States, of the highly-profitable and politically-advantageous rhetoric of damsel in distress.

## Introduction: coloniality of humanitarian and sport superpowers

In this review, we aim to overview (with the intent to make known and better understand) the relationship between sport and global campaigns (and well-intended celebrities, e.g., Ashton Kutcher, Liam Neeson, Demi Moore) that traffick in fantasies of labour abuse, exploitation, and trafficking of vulnerable people—particularly racialized women and gender and/or sexually diverse people in semi-legal economies (such as sex work) or with precarious status in host countries.^1^ Our analysis benefits from studies done on the coloniality of the global anti-trafficking movement—that is, studies which recognize the racialized effects and collateral damages of humanitarian “rescue” industries (see especially 1, 2)—as well as research done on sport mega-event security and surveillance (3, 4). Critical scholarship on the sport mega-event frequently examines security investments in host communities, yet seldom does this work engage with its gendered effects or de/anti-colonial perspectives, especially as related to interventions of humanitarian aid and rescue. We draw from these two overlapping fields to observe the emergence of global “panics” on human (sex) trafficking in mega-event cities as an opportunistic collision and collusion of sentiments of rescue with fantasies of fair play. The partnering of these logics produces an incredibly lucrative and now global movement of synonymous campaigns and non-governmental organizations (NGOs) that caravan with sport (FIFA, the IOC, and Superbowl) and detract from the lived realities of austerity, precarity, and absence of needed labour protections whilst offering a humanitarian slant to the heightened policing and surveillance observed in host contexts.

Few studies interrogate and critique the coloniality of the sport mega-event (see, for exception, 5–7), or the coloniality of anti-trafficking (see, for exception, 1, 2). Often credited as projects of modernity, the sport mega-event and the global anti-trafficking movement are constitutive of coloniality. Peruvian sociologist, Aníbal Quijano, introduced the concept of coloniality and added the caveat that coloniality is only half of the story; the other half is modernity (8). Building upon his work, coloniality is often used to refer to the control and management of knowledge by “universals” of Western modernity, Eurocentrism, and global capitalism (9). Knowledges that are produced as apolitical or neutral, function as means for domination and structure difference in terms of inferiority and superiority. In “Coloniality and Modernity/Rationality,” A. Quijano famously writes,

Coloniality, then, is still the most general form of domination in the world today, once colonialism as an explicit political order was destroyed. It doesn't exhaust, obviously, the conditions nor the modes of exploitation and domination between peoples. But it hasn't ceased to be, for 500 years, their main framework. The colonial relations of previous periods probably did not produce the same consequences, and, above all, they were not the corner stone of any global power. (10).

In his later work, Quijano (11) more explicitly acknowledges the hegemony of capital—and ultimately capitalism—as deeply associated with and connected to coloniality. We emphasize this thought now as dehumanizing forces in the Global North, which naturalized slavery and the application of inhumane labour forms in global capitalism, become relaunched in FIFA/Olympic colonies vis-à-vis “modern slavery” rescue. To observe this trend, we chronologize the emergence of anti-trafficking discourses and strategies in FIFA host cities with a focus on cities that hosted after 2006. We start with the 2006 FIFA World Cup hosted in Germany, which followed Athens, Greece, host of the 2004 Summer Olympics. Greece was the first host nation to act on circulated concerns of women and children trafficked for the purposes of the sport mega-event.^2^ This followed the establishment of the United Nations, *Protocol to Prevent, Suppress and Punish Trafficking in Persons Especially Women and Children* (the Palermo Protocol) which was adopted in November 2000 and entered into force on 25 December 2003. At the time, elected authorities conceptualized the illusive term “human trafficking” as specifically “sex trafficking” or “forced prostitution” and sought strategies to mitigate and further regulate sex workers. This mirrored the actions of the US State Department's Office to Monitor and Combat Trafficking in Persons, which issues an annual report, since 2001, that ranks countries, and guides programs and funding for human trafficking worldwide and calls for funding recipients to denounce prostitution. Despite unfounded fears of increased sex trafficking in 2004 Athens—in fact, the International Organization for Migration (IOM) concluded that there was no relationship between the Olympic Games and human (sex) trafficking (12)—media and authorities speculated that, in Germany, an estimated 40,000 women and children would be sex trafficked for the 2006 FIFA World Cup (see especially, 13, 14).

We are especially motivated to engage in this type of work due to the racist and (hyper)sexualized fantasies that circulated prior to the 2014 FIFA World Cup and 2016 Summer Olympics, both hosted in Rio de Janeiro, Brazil. Such fantasies perpetuate colonial imaginaries of women from the Global South, within a symbolically saturated landscape of racialized (hyper)sexuality, to promote Rio de Janeiro internationally, but also control the bodies and movement of women entangled in representations. This is problematic because provocative fantasies help to promote a sexualized urban realm, suggestive of a racial democracy and cross-class conviviality, yet deny opportunities for women to author their own bodies and sexualities. Fantasies mediate access of women to their own bodily autonomy and agentic capacities (15–18) and, irrespective of their involved in sexual commerce, demand constant vigilance and performances of (white-feminine) respectability. This is the exact terrain in which human rights discourses intervene to traffick carceral logics vis-à-vis feminist benevolence—see especially Elizabeth Bernstein (19–21) and her work on carceral feminism. Feminist sociologist, Elizabeth Bernstein, criticized the contemporary anti-trafficking movement in the United States for its erosion of women's rights, particularly the rights of women in sex work, bolstering state/police powers, and expanding neoliberal agendas through the guise of feminist benevolence. She argues that mainstream feminism has become a vehicle—not for rescue—but of punitive politics in the US and abroad.

Race and racialization are inherent to carceral (feminist) logics. As a framework for our writing, we found the activism and scholarship done in the realm of the police and prison abolition movement to be extremely helpful—particularly in its attention to the racist (as well as sexist, transphobic, whorephobic, ableist, etc.) dimensions of punitive politics. The police and prison abolition movement calls attention to the harms of criminalization and the supposed obligation of the state to control and manage its populace, and instead advocates for the decriminalization of migration (at borders) and survival (within cities, whether related to labour or habitation). We believe that the work of police and prison abolitionists should thereby be taken seriously in critical scholarship on the sport mega-event and future event planning, particularly as events increasingly become opportunities for host nations to showcase their latest in military artillery and police surveillance technologies. Furthermore, in terms of our involvement in sex worker rights activisms, we found the call to decriminalize sex work and cross-border migration as universally advanced by sex worker unions and collective organizations across all the countries discussed in this review. Their advocacy is empirically-driven and although not the focus of this review does necessarily inform our approach to the literatures. We thus logically follow the calls of women and communities directly involved in sex work—the target of anti-trafficking policing and campaigns—to provide the theoretical framework for our analysis and critique.

Integral to our theoretical framework are the contributions of one prominent leader of the contemporary prison abolition movement, Ruth Wilson Gilmore. As a critical geographer, Gilmore is particularly curious and critical of spaces and practices of incarceration, and most often credited and referenced in relation to the concept of carceral geography (see especially 22). We maintain that her work is especially needed to understand the relation between sport and humanitarian superpowers—and the control and containment of carceral subjects (prisoners, detainees, refugees, etc.) in contemporary times. Specifically, Gilmore (23) offers the term “shadow state” in reference to the “non-profit industrial complex” to acknowledge the expansion of the non-profit sector and its links to diminishing state welfare, aid, and investments. If, on the one hand, economic and political priorities are increasingly directed to/by national security, the intellectual and material landscape—through the establishment of bases, facilities, expertise—will forever be transformed. This transformation establishes the “prison industrial complex”, which conceptually accounts for the decisive focus on foreign and domestic punishment, and the hidden cost on communities doubly debilitated by punishment and negligent social care. The point is not that few companies dictate realities—Gilmore (23) is abundantly clear on this point, and we aim to emphasize it yet again—but that an entire realm of social investments are held hostage to the development and perfection of the prison industrial complex, which in turn normalizes the belief that aggression is key to safety. She continues her argument to observe the immense investment in the prison industrial complex as rationalizing the emergence of entities (neither state nor business) that replace, supplement, even duplicate government agencies. Government agencies previously existed with the mandate to attend to those most in need and now exist as bureaucracies which manage services and govern through policing efforts. Gilmore then theorized the emergence of the “non-profit industrial complex” in response to organized state abandonment. Rather than operate in a vacuum, the non-profit sector is largely forced to reflect the mandates and demands expressed by governments in order to receive financial support. The “shadow state” is thus without significant political clout and prohibited to advocate for systemic change. The association of the allegedly “non-profit” (yet super profitable) sector ultimately affords the sport mega-event added opportunities for conflicting political logics to congeal and ascertain global recognition vis-à-vis rhetorics of rescue. All whilst overly simplified, individually-minded remedies are propagated that fail to acknowledge, but maintain structures and circumstances of violence, which sport and humanitarian superpowers then benefit from.

## Trafficking (in) securities: FIFA and fantasies of rescue

We came at this review through the perspective that sport mega-event investments should not reify forms and structures of neoliberal racial capitalism—and aspire for the same to be true for the global anti-trafficking movement. We trace the responses to human (sex) trafficking in all FIFA host contexts, since the first trafficking “panics” were associated with the male-dominated football/soccer event in 2006 Germany. In all host contexts, governments and NGOs devised responses that focused on individual choices, prescribed individualized solutions, rather than attending to the structures that condition precarity into daily life. Despite the absence of victims, anti-trafficking “activism” has grown into a global movement that amasses millions in revenues and celebrity endorsements for organizations in the allegedly non-profit sphere (24). To this end, anti-trafficking campaigns perpetuate the violence racialized women and gender-diverse people disproportionately face in the dominant colonial-capitalist world system. We provide an overview of the separate yet connected anti-trafficking efforts and rhetorics of rescue in FIFA host cities more thoroughly now. Noting that moral panics seem particularly fixated on women in heterosexual sex industries, and much of the literature relatedly prioritizes women sex workers with men as clientele.

### 2006 Germany

Prior to the 2006 FIFA World Cup hosted in twelve cities across Germany, there was considerable international concern that the event would create a sharp increase in trafficking for sexual exploitation. Zita Gurmai, a Hungarian politician at the time, expressed concern in a statement to European Parliament: “from past experience—for example in Athens, during the Olympics—we have seen that international sporting events cause an increase in human trafficking” (25). European Parliament soon enlisted support for several national entities to launch a Europe-wide prevention and education program and to ratify the Council on Europe Convention on Action Against Trafficking in Human Beings. The US also extended their efforts to endorse strategies which serve to pressure elected authorities in host countries to criminalize prostitution (26). US Congressman Christopher Smith sponsored a resolution to encourage the German government to implement the Palermo Protocol and the US State Department's Office to Monitor and Combat Trafficking in Persons recommended the German government to increase police enforcement for the duration of the World Cup.^3^ NGOs requested support from the German government to orchestrate hotlines, shelters, and public campaigns. Four major campaigns emerged at that time, and included, Red Card to Sexual Exploitation and Forced Prostitution, Final Whistle: Stop Forced Prostitution, Stop Forced Prostitution, and Action Against Forced Prostitution. The German government also agreed to increase police presence in host cities and develop a national prevention plan. In the end, despite the increased level of concern and surveillance, no marked increase in human trafficking occurred in Germany during the World Cup (27–29). In fact, a report commissioned by the International Organization for Migration (IOM) determined that “all data, information and expert statements that are available to date strongly indicate that an increase in human trafficking did not occur either during or after the World Cup” (27 p. 5). Nevertheless, unfounded fears continued to mobilize “rescue” industries through discourses of health and contagion at the 2010 World Cup.

### 2010 South Africa

In South Africa, the rhetoric of women and children trafficked for the sport mega-event amplified another facet of sexual panic. The migration of women and children was imagined from other African countries, which amplified familiar punitive and highly racialized strategies within the nine host South African cities and, especially, at the national border. At the same time, international media amplified public health concern, which fixated on HIV transmission to limit or dissuade sex tourism (30, 31).^4^ Despite increased surveillance and related police violence: there was zero increase in people trafficked for the event nor increase in HIV transmission (30, 36, 37). Richter et al. (30) note a total lack of evidence to demonstrate an increase in migration of women and children. Whereas women already involved in sex work within host cities reported a decrease in business (i.e., reduction in clientele despite influx in tourism and associated decrease in women at work in the month). These realities challenged the claim that women, particularly young women, enter the profession for the duration of the sport mega-event (27) as well as the assumption that an increase in tourism would subsequently increase business. While some women reported benefit, this was not common—in fact, only a third of women commented on a change in typical business and the median amount charged per client remained unchanged (30, 38). The heightened attention and financial investment directed at anti-trafficking strategies—particularly those that targeted women in sex work—remained unsubstantiated and instead fed public perceptions that women engaged in sexual commerce are diseased and trafficked without critique of broader socioeconomic dynamics that contribute to illness, disease, and violence. We continue to see health weaponized against women in sex work as demonstrated with Rio de Janeiro as international concern related to the Zika virus erupted prior to the 2016 Summer Olympic Games (see also 39).

### 2014 Brazil

**Figure 1:**
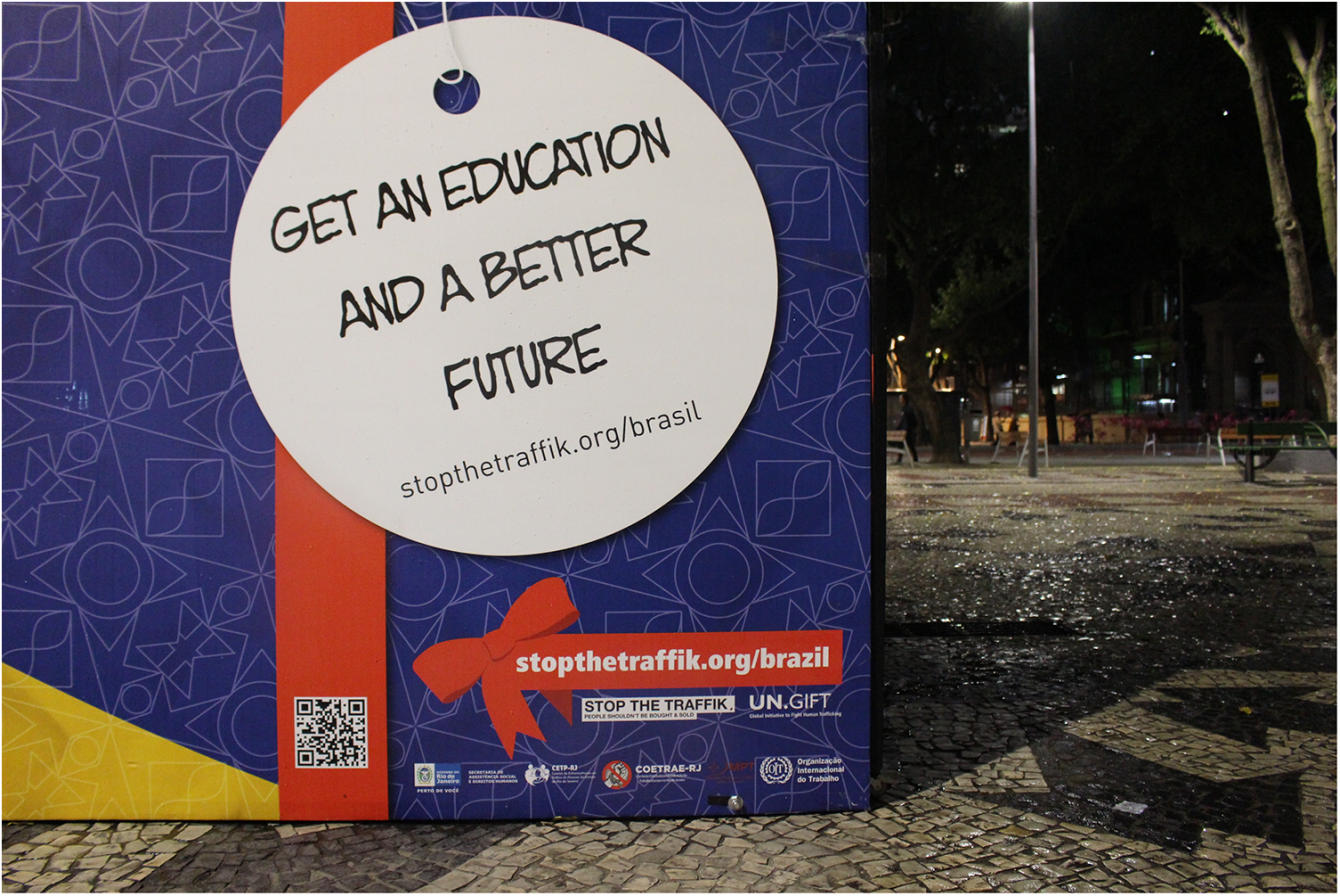
UN Gift Box in Centro (downtown), Rio de Janeiro. The UN Gift Box (Global Initiative to Fight Human Trafficking) is a public art installation strategically positioned within FIFA/Olympic host communities to draw attention to and metaphorically mimic the way in which a trafficker might entice and entrap their victim. The inside of the box is lined with testimonial stories from people allegedly trafficked. Photographed by Amanda De Lisio.

**Figure 2:**
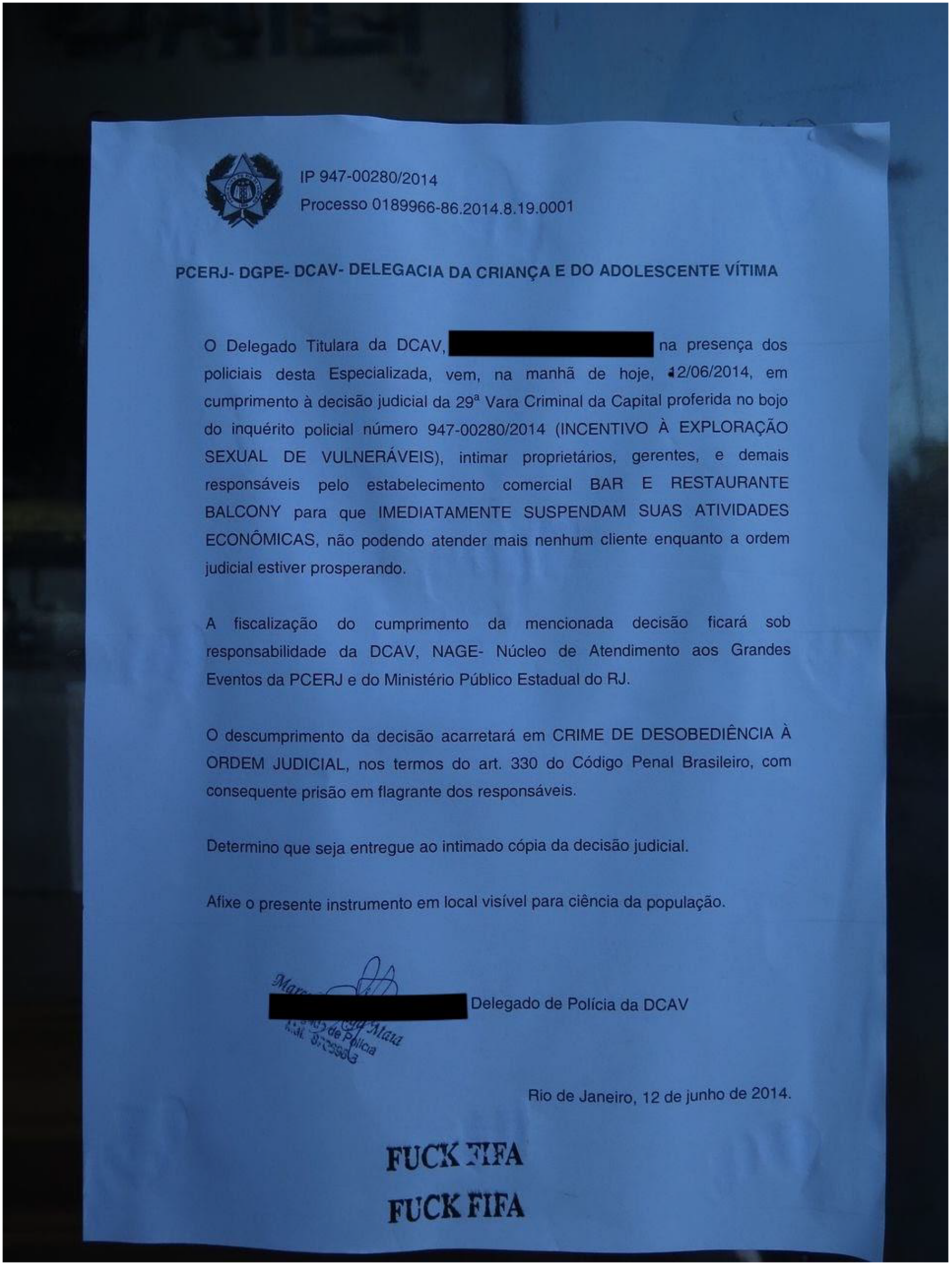
Public notification posted to a highly-visible beachfront restaurant in Copacabana on the day of 2014 FIFA world Cup Opening Ceremony (12 June 2014). The notice called for the immediate suspension of all commercial and economic activities due to an allegation of child sexual exploitation. No formal charge or arrest was ever formally made, and the business was forced to close, permanently, soon thereafter. Photograph by Amanda De Lisio.

**Figure 3:**
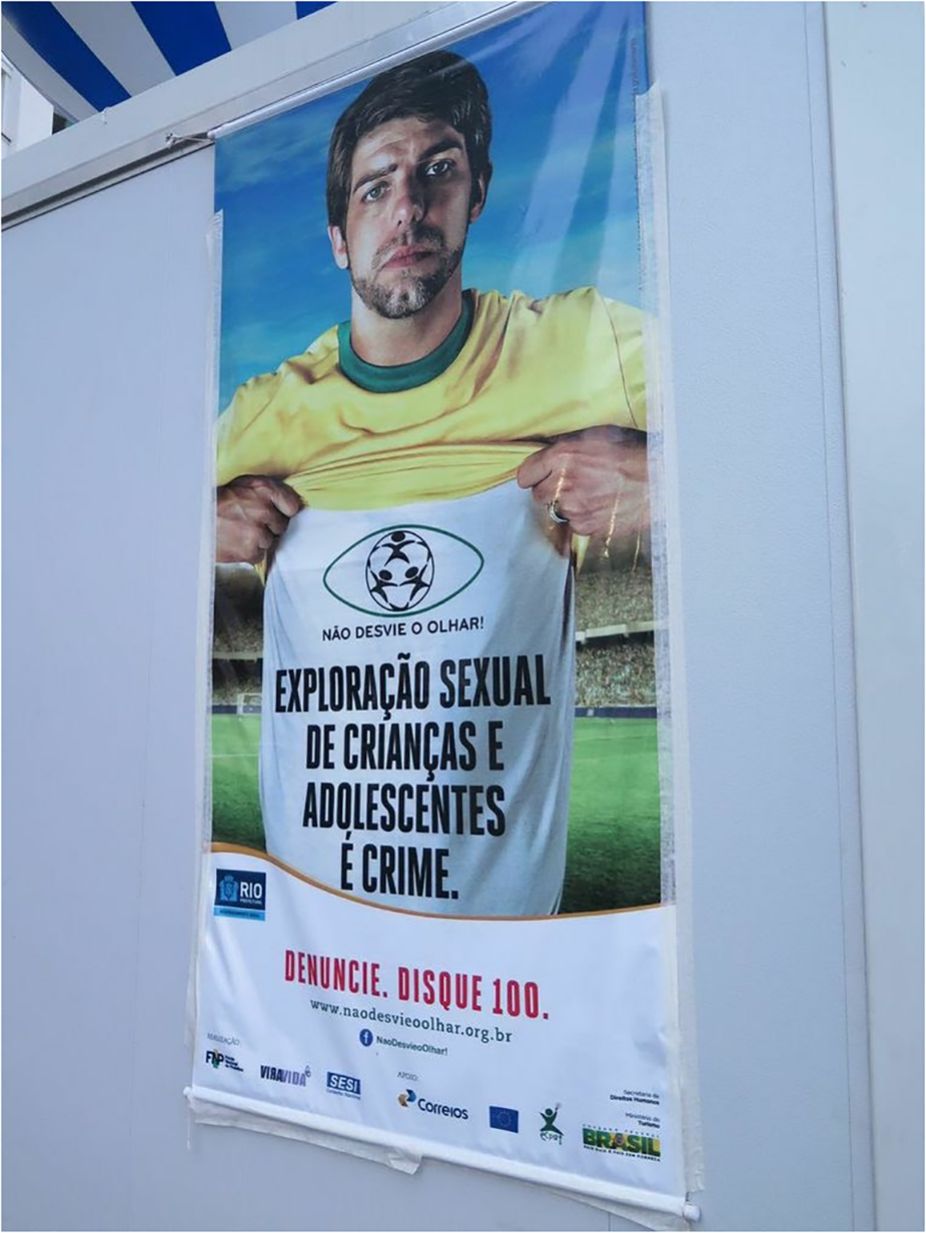
Campaign to advertise and attract attention to the potential for child (sexual) exploitation posted in copacabana beach. This poster advertises a hotline that local people could call to denounce a crime, Disque 100. Photograph by Amanda De Lisio.

**Figure 4:**
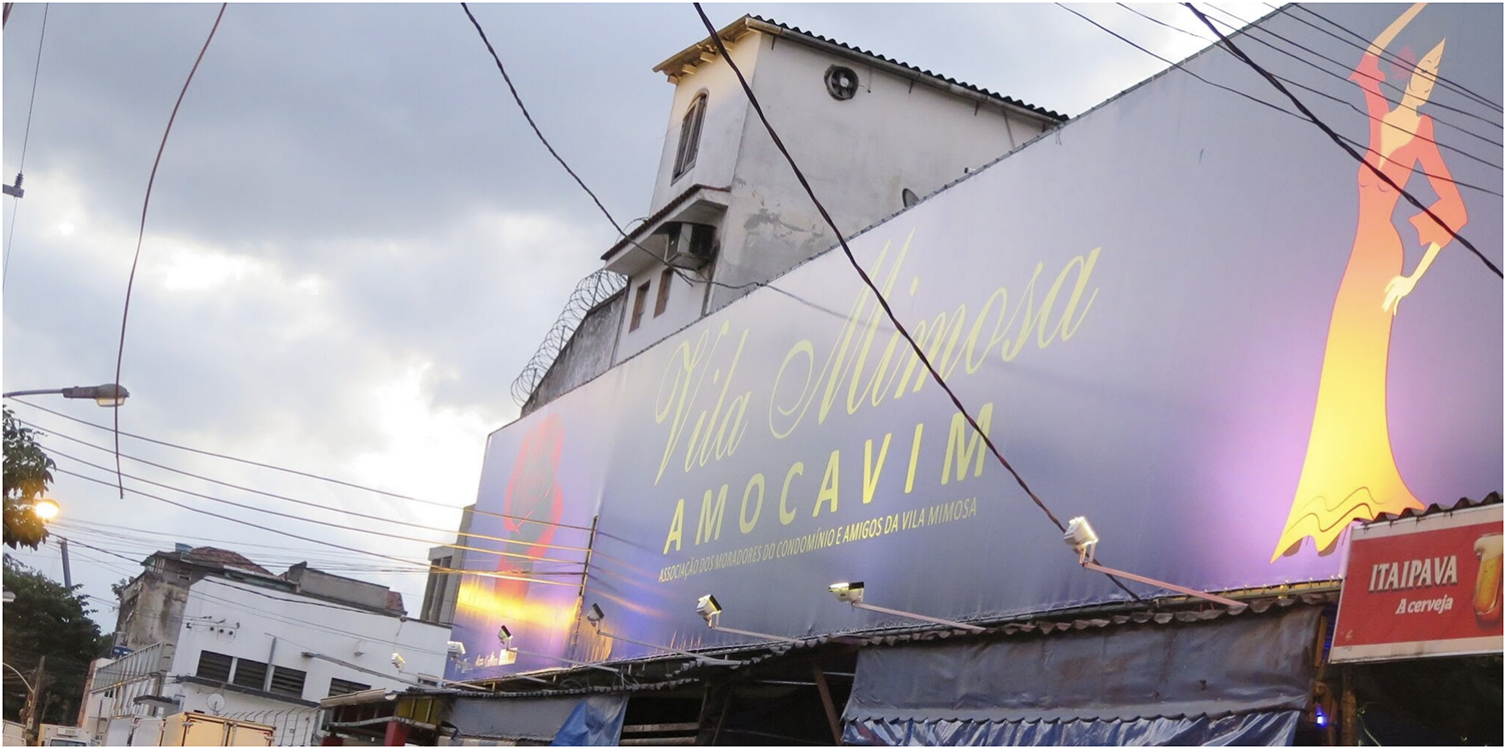
Sign posted to advertise the local sex worker union in Vila Mimosa, the red-light district in Rio de Janeiro. The union organized to upgrade the area ahead of the 2014 FIFA World Cup; mindful that businesses were less than a kilometre from Maracanã Stadium, and optimist that the increase in tourism would attract additional clientele. Photograph by Bryan Clift.

For the 2014 FIFA World Cup hosted in several cities throughout Brazil (namely, Rio de Janeiro, Brasília, São Paulo, Fortaleza, Belo Horizonte, Salvador, Porto Alegre, Recife, Cuiabá, Manaus, Natal, and Curitiba), the federal government increased investment in national as well as more coordinated and localized anti-trafficking efforts. This was combined with international agencies and campaigns, such as the UN and their Gift Box campaign (see Figures 1). Again, this occurred without the consultation of women historically involved in sex worker activism in Brazil—celebrated for the recognition of sex work as an official profession within the Brazilian Ministry of Labour and Employment (CBO 5298–5) in 2002, which affords access to social security via the Ministry of Social Security and the National Institute of Social Security (INSS), and internationally acclaimed for their approach to activism which centres on the harmful role of stigmatization and criminalization. In 2005, Brazil denounced the US Agency of International Development “anti-prostitution pledge” and the associated $48-million in funding for HIV prevention. Despite such advancements, humanitarian efforts remained focused on women in sexual commerce as victims, and positioned the Gift Box in Vila Mimosa (the red-light district in Rio de Janeiro, located less than a kilometre from Maracanã Stadium). Importantly, Vila Mimosa is a neighbourhood already familiar with police brutality, twice displaced from the downtown core via processes of modernization that appealed to international classes and attendant capital (40, 41). Of such efforts, less attention was directed at the illegal police raid in Jardim Itatinga, a red-light district in Campinas, in the State of São Paulo, considered the largest confined urban zone for prostitution in Latin America (42). Jardim Itatinga was erected amid the military dictatorship in a brazen attempt to isolate prostitution, otherwise thought to threaten social order. Prior to the World Cup, police rummaged warrantless throughout the neighbourhood, threatened, and tortured those involved in sexual commerce and, in one particularly violent incident, intentionally broke the arm of a Colombian sex worker (43). There was likewise failure to find or leverage adequate support for the more than hundred women raided, evicted, and permanently displaced from their autonomous places of employment in Niterói, an area of increased real estate speculation, amid mega-event reconstruction. Women reported rape, theft, extortion, and violence but local police were never held accountable (44, 45). The Public Defender assigned to the case declared the raids “completely illegal” and “driven by the stigma” against sex work. Similar fantasies of (child) sexual exploitation were mobilized on the day of the 2014 FIFA Opening Ceremony, with the closure of a beachfront restaurant, close to the FIFA Fan Fest in Copacabana. The business was declared a site of child sexual exploitation and forced to permanently close even as no arrest and/or formal charge was ever made (see Figure 2). In fact, in 2014, not a single case of child sexual exploitation was brought forth for litigation to the State of Rio de Janeiro (46). In 2016, one incident of child sexual exploitation involved twelve men that were arrested and convicted and included a municipal councillor and federal deputy from the military police (47), another involved a military police coronel (48). These egregious felonies were unrelated to the sport mega-event and did not involve a single foreigner. Most harmed in the process were those already most vulnerable, particularly migrant women with precarious status, fearful of deportation, and trans* communities, most often the target of murderous violence in Brazil. With campaigns to attract attention to child (sexual) exploitation in Brazil, local women were often made the target of humanitarian aid and law enforcement; despite their effort to organize, renovate and better advertise their businesses (see Figures 3, 4). Similar to other FIFA host cities, women involved in the diverse industries of sexual commerce throughout Rio de Janeiro reported little impact of the FIFA World Cup on their business activities (49).

### 2018 Russia

While previous FIFA host countries introduced strategies to curb the presumed influx of forced (sexual) labour, the Russian government did not sponsor any campaigns aimed at the eradication of trafficking prior to the 2018 FIFA World Cup (50). This caused outrage across international communities, many of whom feared an increase in forced prostitution in Russia, particularly amidst a newly-introduced laxed visa regime (51). Prostitution is illegal in Russia, yet financial penalties associated to prostitution fail to deter people from the profession, and people instead maintain a parallel network of informal and semi-legal allies—in the shadow of the shadow state (to borrow from 23, 52). It is estimated that roughly one million people are involved in sex work in Russia (53). Prior to the 2014 Winter Olympics, Russia tightened legislation related to prostitution, which sought to severely punish (even imprison) people in sex work. With the 2018 FIFA World Cup, Russia issued a new decree to strictly control prostitution in the eleven host cities: Samara, Nizhny, Novgorod, Volgograd, Ekaterinburg, Saransk, Rostov, Kaliningrad, Kazan, Moscow, Sochi, and St. Petersburg. This was linked to the federally mandated “Safe City” program, introduced at the same time Russia bid to host the 2014 Winter Olympics and 2018 FIFA World Cup, which focused on the public presence of alleged social deviance as a cornerstone of urban security and named prostitution as a key target of the program (54). The program allowed for the implementation of aggressive surveillance technologies within host cities, which particularly targeted communities of alleged threat (55). The militarization of host cities familiarly correlated with an increase in police harassment (i.e., several businesses were forced to close, and women subjected to brutality) as reported by Silver Rose, a national sex worker-led organization (56).

Irina Maslova, director of Silver Rose, reflected on the similar strategies observed prior to the 2018 FIFA World Cup which resembled the strategies prior to the 1980 Summer Olympic Games, hosted officially in Moscow wherein she and other marginal people were violently moved beyond the boundaries of the centre, away from tourist view. With harsher legal penalties related to the profession, and associated harassment from law enforcement, women (primarily) and gender-oppressed people autonomously involved in sexual commerce experienced diminished opportunities to benefit from the influx of male tourism—combined with a lack of harm reduction and increased cost of contraception. At the same time, local police benefited from their elevated powers to discretionarily and discriminatorily enforce the law and through threat and intimidation were able to force women to perform sexual activities (referred to as “buy-and-bust” operations wherein sexual services are performed for pay as evidence of prostitution) or submit a *krysha* (literally roof or bribe, which is costlier than the actual business fine: e.g., US$80–250 compared to US$25–30) to authorities (56). Whereas women were forced from the tourist view, entrepreneurial men were able to respond to the alleged increase in sexual demand via their continued operation of strip clubs and recently instituted and legalized “Doll Hotels” or sex robot brothels, which charged a client roughly £60 per hour in a private room with a human stimulator (57, 58).

### 2022 Qatar

With the 2022 FIFA World Cup hosted in three cities in Qatar (i.e., Al Khor, Al Wakrah, and Doha) much of the public criticism was directed at labour exploitation and forced migration (see, for example, 59–61)—in the absence of discussions with women and gender-diverse people involved in sexual commerce. Particularly, international media focused on stadium construction to address the kafala (sponsorship) system wherein migrant workers must surrender their passport to obtain and then maintain sponsorship to legally work (62, 63). Renkiewicz (64) discusses the power imbalances typical of the sponsorship system, created to privilege companies in lieu of individual workers and which result in conditions comparable to “modern-day slavery” (p. 725). The emphasis on the rhetoric of modern-day slavery can highlight the continuation of colonialism and attendant processes of racialization that are necessitated and enshrined within capitalism. South and Southeast Asian migrant communities are repeatedly targeted in recruitment strategies and then isolated and segregated from one another upon arrival (65). Despite the recent effort to reform this system, people still express difficulties with vigorous implementation and the enforcement of new labour legislation (see, for example, 66). A campaign entitled, #PayUpFIFA has recognized the need for labour reparations; particularly for families of those deceased in construction. Less emphasis is directed at sexual exploitation or forced prostitution due to the stringent legislation in the nation. As written in Qatar legislation (via Article 298 of Law No. 11, 2004): “whoever performs adultery or sodomy as a profession or for a living shall be punished with imprisonment for a term up to ten years. The same penalty shall be imposed on any person who exploits another person's immorality and prostitution” (as cited in 67, p. 232). This has made sex work, typically orchestrated within areas known for tourism (e.g., the lobby or nightclub affiliated of a Western hotel) as mostly engaged by migrant women without Qatari citizenship, subject to egregious criminalization. Gender-based violence in the form of sexual assault and labour exploitation is often unreported as related to prostitution and instead observed and documented in the specified case of domestic labour wherein women report heightened vulnerabilities to rape, sexual exploitation, etc. (68, 69). We are thankful that conversations in/on Qatar shifted slightly from unfounded fears of women and children trafficked for sexual exploitation for the duration of the World Cup, and instead aimed to further appreciate the complexities of the problems/solutions anti-trafficking campaigns allege to address/provide through hotlines and awareness campaigns.

## Conclusion: beyond the coloniality of rescue and the sport mega-event

Through our review of anti-trafficking discourses and strategies in FIFA host cities, since 2006, we hoped to add to further conversations on the coloniality of anti-trafficking and the sport mega-event. We observe the global anti-trafficking movement as an extension of carceral logics that—through feminist benevolence—heightens surveillance and attempts to further control the bodies, sexualities, and needed economic opportunities of all women in host contexts, particularly racialized and trans* women, but is especially harmful on sex workers. We encourage future strategies to engage directly with women and gender-oppressed people with whom such strategies are alleged to most aid and listen to their experiences and adhere to their lived and living expertise. There is, despite the belief that sex work is forced to the shadow and repressed within world-class cities, no shortage of sex worker solidarities and resistance activities locally organized and prepared to contest familiar forms and structures of neoliberal racial capitalism, such as those reified by the sport mega-event. As Irina Maslova made abundantly clear in her account of the 2018 FIFA World Cup preparation in Russia—despite the appeal to modernity—the sport mega-event remained a relic of historic urban struggle, an inevitable consequence of colonial-capitalist development. Rather than react to the unfounded and unsubstantiated claim that women and children are trafficked for the sport mega-event, it is time for those that caravan with the sport mega-event to understand histories of geopolitical development and the lived consequences for marginalized communities with whom they intend to aid.

Whereas other forms of labour exploitation and abuse (e.g., with young athlete recruitment, sport equipment and athletic apparel, and construction) are routinely sidelined, we too focused on the particular assemblage of expertise mobilized via unfounded, highly sexualized, gendered, and racialized anxieties related to “women in (forced) prostitution” and the type of feminist solidarities necessitated in response. In turn, we observe the opportunistic culture created through moral policing and campaigns to end human (sex) trafficking, which rationalize immense investments in event securities and create gendered insecurities—i.e., criminalize and control migrant women, gender-diverse people, and those perceived to be or actually involved in sex work or adjacent industries (e.g., alternative massage). Human trafficking is commonly conflated with, and increasingly used as a tandem spectacle to the sport mega-event to target racialized, hypersexualized women (cis and trans*) in semi-legal economies or with precarious status. Rather than further conflate sex work with human (sex) trafficking, we wish to keep at the forefront of future research, the highly specified and contextually situated circumstances that do not focus on individuals engaged in supposedly reprehensible or immoral labour practices, but instead strive to unveil the incredibly lucrative economies that sustain racialization, violence, and enshrine dangerous labour conditions.

Economies of alleged care emerge to “rescue” women from supposedly coercive, forceful, and/or exploitative labour, without reference to the fact that much of the legal labour opportunities made available to low-income, racialized women in late capitalism often involve some variation and/or level of coercion, force, and exploitation. It also denies the ways in which sex work is similar to sport work, which can at times be exploitative and damaging, but is not always the sole or only outcome of the profession. Through the sport mega-event then, and more specifically through FIFA World Cup host cities, we observe a unique cascade of state and non-state authorities, who actively target and intervene with women in movement/migration and feminized (sexual) labour (e.g., directly through border patrol, travel and tourism companies, airlines, etc. that campaign against human trafficking and deputize the public to scrutinize racialized women at work and in transit). The migration of anti-trafficking processes, policies, and personalities, which restrict the movement of women and gender-oppressed people (particularly those racialized as non-white) within cities and across borders, maintain the status quo. We urge future advocates and authorities in host cities to instead focus on protecting the networks of highly localized yet globally attuned feminist collectives that foster more humane economic realities and forms of survival in mega-cities—not those that traffick in fantasies of their precarity and vulnerability.

## Footnotes

^1^We use the term semi-legal to discuss sex work which, dependent upon the host nation could be legal per se, but often activities related to the act (adult, consensual sex in exchange for money) are criminalized. The ambiguous nature of prostitution legislation has afforded power to law enforcement, whom discretionarily (and often discriminatorily) decide the law on a circumstantial basis and fuel the call for decriminalization as articulated by sex worker rights organizations, internationally.

^2^Albeit in Athens, Greece, host of the 2004 Summer Olympic Games, it was popularly alleged within international media that local authorities encouraged sex tourism and used the increase in permitted businesses as evidence of heighted possibilities for commercial sex. However, KAGE, a sex worker union in Greece, argued that businesses were already permitted, and legal, but local authorities added regulation prior to the Olympics, which required businesses to obtain an additional or new permit.

^3^Press Release, German Embassy, Washington D.C., Fighting Human Trafficking: Measures Taken by Germany in Relation to the Soccer World Cup (June 10, 2006). Available at https://chrissmith.house.gov/news/documentsingle.aspx?DocumentID=56013 (last accessed May 9 2023)

^4^Migrant women and women in sex work are often pathologized; despite their leadership in sexual health and public health intervention—this is particularly salient in histories of HIV/AIDS prevention, and undeniably reinforced in the immediate community-based response to COVID-19 (see, for example, 32–35). Yet the rhetoric of sex worker as contagion is used to discriminatorily target racialized women and gender/sexual diverse people as diseased and thereby stigmatized and subjected to routine and mandatory medical examination.
